# A comparison of the exposure system of glycidol‐related chemicals on the formation of glycidol‐hemoglobin adducts

**DOI:** 10.1002/fsn3.3770

**Published:** 2023-10-30

**Authors:** Yuko Shimamura, Yuri Wada, Moeka Tashiro, Hiroshi Honda, Shuichi Masuda

**Affiliations:** ^1^ School of Food and Nutritional Sciences University of Shizuoka Shizuoka Japan; ^2^ R&D Safety Science Research, Kao Corporation Tochigi Japan

**Keywords:** 3‐monochloropropane‐1,2‐diol, epichlorohydrin, glyceraldehyde, glycidol, hemoglobin adduct

## Abstract

Glycidol fatty acid esters that are present in foods are degraded in vivo to the animal carcinogen glycidol, which binds to the *N*‐terminal valine of hemoglobin (Hb) to form *N*‐(2,3‐dihydroxypropyl)valine (diHOPrVal) adducts. The existence of other chemicals that are converted to glycidol is unknown. To determine the effect of different exposure conditions on the formation of diHOPrVal adducts, several glycidol‐related chemicals (3‐monochloropropane‐1,2‐diol; 3‐MCPD, epichlorohydrin, glyceraldehyde, acrylic acid, and 1,2‐propanediol) were evaluated using in vitro and in vivo (single/repeated dose) methods. In vitro, the reaction of 3‐MCPD or epichlorohydrin with human Hb produced 17% and 0.7% of diHOPrVal, as compared to equimolar glycidol, respectively. Following a single administration of glycidol‐related compounds to ICR mice, diHOPrVal formation was observed only in the epichlorohydrin‐treated group after day 5 of exposure. After 14 days of repeated dosing, the amounts of diHOPrVal produced by epichlorohydrin and 3‐MCPD in vivo were <1% of diHOPrVal produced by an equal molar concentration of glycidol. Furthermore, glyceraldehyde group produced 0.2% of diHOPrVal at the same molar concentration of glycidol equivalents, in which diHOPrVal formation could not be confirmed by the in vitro assay. The results indicate the usefulness of diHOPrVal as an exposure marker for glycidol; however, the contribution of its formation in vivo by exposure to various chemicals will be necessary to validate and interpret the results.

## Graphical abstract

The exposure systems of glycidol‐related chemicals on the formation of glycidol‐hemoglobin adducts diHOPrVal were compared. DiHOPrVal formation was observed using several chemicals other than glycidol with different behaviors in vitro and in vivo. However, in both cases, the amount of diHOPrVal produced was very small compared with glycidol. This study confirmed the usefulness of diHOPrVal as an exposure marker for glycidol, although further study of the contribution to its formation by the in vivo behavior of each chemical will be useful in interpreting the results.

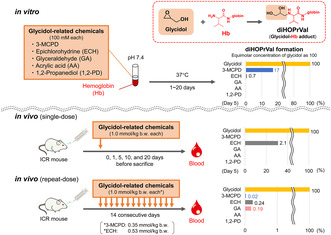

## 1 INTRODUCTION

Glycidol fatty acid esters (GEs) are contaminants that form during deodorization processes under high temperature during the production of edible oil. Relatively high concentrations of GEs have been detected in products containing refined vegetable oil including infant formula (Bakhiya et al., 2011). In food products, GEs are also formed during heat treatment of fish and meat patties, especially at high temperatures during charcoal grilling (Inagaki et al., 2016, 2019), and in commercially prepared foods, such as instant noodles, fried chicken, and fried confectionery (Shimamura et al., 2021). GEs present in foods are thought to be degraded in vivo into the animal carcinogen glycidol by lipases. Glycidol can bind to the N‐terminal valine of blood hemoglobin (Hb) to produce a glycidol‐Hb adduct (*N*‐2,3‐dihydroxypropyl)valine: diHOPrVal) (Landin et al., 1996), which may be used to determine glycidol exposure (Honda et al., 2011, 2012, 2014; Rietjens et al., 2018). Hb adducts of chemicals are considered long‐term exposure indicators because they accumulate in the body over the lifespan of human erythrocytes, which is approximately 120 days (Shemin & Rittenberg, 1946; Troester et al., 2000). In vivo studies using rats confirmed the dose‐dependent production and chemical stability of diHOPrVal (Honda et al., 2014). When 11 healthy subjects ingested approximately 36 g of commercial palm oil (8.7 mg glycidol/kg) daily for 4 weeks, the average daily glycidol exposure estimated from the adduct level (background level) before intervention was 0.94 g/kg b.w (Landin et al., 1997). The estimated daily glycidol exposure in 50 12‐year‐old children, as measured by diHOPrVal levels, was 1.4 μg/kg/day (Abraham et al., 2019). These values were higher compared with the estimated intake of glycidol for adults and children calculated by the European Food Safety Authority (adults: 0.2 μg/kg/day, children: 0.6 μg/kg/day (Aasa et al., 2019)), which showed no relationship between the intake of GEs and the amount of diHOPrVal. These findings suggest that other chemicals besides GEs produce glycidol in vivo and human may be constantly exposed to them.

Several chemicals have been proposed as possible precursors for diHOPrVal formation and their putative formation pathways. For example, 3‐monochloropropane‐1,2‐diol (3‐MCPD) fatty acid esters (3‐MCPDEs) are considered precursors of diHOPrVal (EFSA panel on contaminants in the food chain (CONTAM), 2016). GEs themselves are also produced from 3‐MCPDEs under neutral, acidic, and alkaline conditions (Cheng et al., 2017). Content in foods prepared by heating in the presence of frying oils/fats of 3‐MCPDEs were in the range of 0.1–0.5 mg/kg, with the highest levels in doughnuts (1.2 mg/kg) and French fries (6.1 mg/kg). (Bakhiya et al., 2011) Although epichlorohydrin was mostly considered an occupational exposure, diHOPrVal was detected in the blood of subjects without any occupational exposure to epichlorohydrin (Aasa et al., 2017). Epichlorohydrin is used as a wetting and strengthening agent in the manufacture of cellulose products such as coffee filters and tea bag paper, and tea leachate may contain epichlorohydrin and/or its metabolite 3‐MCPD. Estimated daily intakes from tea bags have been reported to be 1.03 × 10^−2^ mg/kg/day for epichlorohydrin and 1.00 × 10^−6^ mg/kg/day for 3‐MCPD (Nour et al., 2023). The Hb adduct of glyceraldehyde can be converted to diHOPrVal under reducing conditions (Landin et al., 2000). Glyceraldehyde is a metabolic intermediate of fructose, a natural sugar, in vivo. Glyceraldehyde content in human plasma is about 2–20 μM (1.8 × 10^−4^–1.8 × 10^−3^ mg/kg). (Martin‐Morales et al., 2021) Allyl alcohol is produced when garlic is heated at 121°C (Chung et al., 2007; Laakso et al., 1989) and it is metabolized to acrolein by hepatic alcohol dehydrogenase in vivo (Mapoles et al., 1994). In addition, acrolein is metabolized into acrylic acid (Patel et al., 1980), which is used as a raw material for dispersants, flocculants, thickeners, and adhesives (Chan & Chu, 2001), and into glyceraldehyde (Patel et al., 1980), a glycolytic intermediate of fructose (Sillero et al., 1960). Acrylic acid is used as a raw material for a food additive (sodium polyacrylate) and the predicted maximum exposure calculated from food data is reported to be 2 × 10^−7^ mg/kg/day (Daecke et al., 1993). Furthermore, 1,2‐propanediol is used as a moisturizer and emulsifier and is present in e‐cigarettes (Chen et al., 2021). 1,2‐Propanediol is also used as a food additive, and the average daily intake from food in the United States is estimated to be 34 mg/kg/day (National Toxicology Program, 2004). These non‐glycidol chemicals have the potential to produce diHOPrVal under various conditions and in vivo, but this has not yet been experimentally verified.

In our previous study, diHOPrVal was not produced from chemicals other than glycidol or glycidol‐related chemicals (epichlorohydrin, propylene oxide, 1‐bromopropane, allyl alcohol, fructose, and glyceraldehyde), although a single dose of these chemicals was administered to rats (Shimamura et al., 2020). Humans are routinely exposed to these chemicals and it is possible that the amount of diHOPrVal produced is small following a single administration to experimental animals. In addition, they may be converted into other metabolites in vivo, resulting in the formation of Hb adducts other than diHOPrVal. Furthermore, a temporal problem may exist during the conversion of these chemicals to glycidol in vivo.

To more accurately assess the risk of glycidol in food, it is important to determine which chemicals lead to the formation of diHOPrVal, an in vivo exposure biomarker in humans. To understand the influence of in vivo behavior of chemicals, such as absorption, distribution, metabolism, and excretion (ADME) processes, on the Hb adduct formation, this study compared the formation of diHOPrVal from glycidol‐related chemicals using in vitro and in vivo methods. 3‐MCPD, epichlorohydrin, glyceraldehyde, acrylic acid, and 1,2‐propanediol were used as glycidol‐related chemicals. The in vitro studies confirmed the direct production of diHOPrVal from glycidol‐related chemicals. In vivo studies also revealed the generation of diHOPrVal following single and 14 days repeated administration to Institute of Cancer Research (ICR) mice, considering the ADME of the chemical.

## 2 EXPERIMENTAL SECTION

### 2.1 Chemicals

Glycidol (purity 96.0%), fluorescein‐5‐isothiocyanate (FITC purity ≥90.0%), human hemoglobin lyophilized powder, and 1,2‐propanediol (purity 99.5%) were purchased from Sigma‐Aldrich. Acetonitrile and glyceraldehyde (purity 98.0%) were obtained from Tokyo Chemical Industry Co., Ltd., Kanto Chemical Industry Co., Ltd., and Nacalai Tesque, Inc., respectively. Other chemicals (L‐valine, L‐*d*_8_‐valine, sodium chloride, potassium bicarbonate, hydrochloric acid, ethyl acetate, sodium sulfate, toluene, ethyl acetate, ethanol, methanol, ammonium hydroxide, 3‐MCPD (purity 98.0%), epichlorohydrin (purity 99.0%), and acrylic acid (purity 98.0%) were purchased from Wako Pure Chemical Ind., Ltd.

### 2.2 Preparation of diHOPrVal standards

L‐valine sodium solution or L‐*d*_8_‐valine sodium solution (1 mL of 1 M each) was mixed with 74.0 mg glycidol (96% purity), stirred, and heated at 60°C for 18 h. The product was acidified using 2 mL of 1 M hydrochloric acid and extracted with a solution of ultrapure water: ethyl acetate (1:1). The organic layer was washed twice with ultrapure water and dehydrated with sodium sulfate. The dehydrated organic layer was dried under reduced pressure using a rotary evaporator. The dried sample was dissolved in acetonitrile and separated by thin‐layer chromatography Silica gel 60 F_254_, MERCK, Darmstadt, Germany. The developing solvent was toluene: ethyl acetate: ethanol (3:3:1; v/v/v). The spot representing the substance of interest was scraped from the thin plate and the FITC‐derivatized material was extracted and purified using methanol. The resulting samples were dried in a rotary evaporator and used as glycidol‐L‐valine adduct fluorescein thiohydantoin (diHOPrVal‐FTH) and the internal standard DHP‐Val‐*d*_7_‐FTH.

### 2.3 In vitro measurement of diHOPrVal production by the reaction of glycidol‐related compounds with human Hb

**FIGURE 1:**
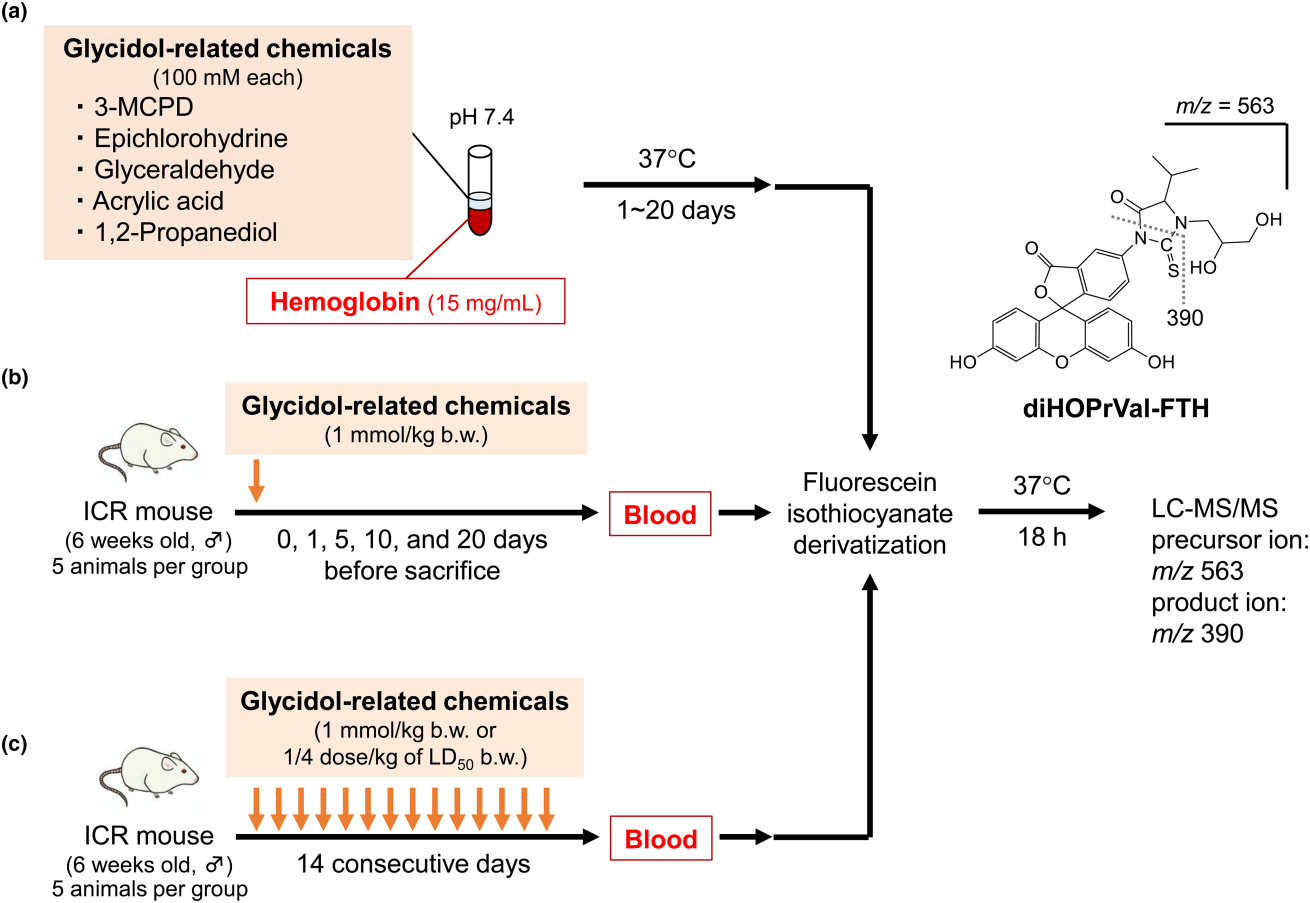
Scheme of this study. (a) Human hemoglobin (Hb) and glycidol‐related chemicals were incubated in vitro to evaluate the formation of glycidol‐hemoglobin adducts (diHOPrVal). (b) ICR mice were administered a single oral dose of glycidol‐related chemicals and the formation of diHOPrVal in the blood was evaluated. (c) ICR mice were given repeated oral doses of glycidol‐related chemicals (once daily for 14 days) and the formation of diHOPrVal in the blood was determined.

Hemoglobin (Sigma‐Aldrich) was dissolved in 0.1 M phosphate buffer (PB) (pH 7.4) to 30 mg/mL. Each chemical (glycidol, epichlorohydrin, 3‐MCPD, glyceraldehyde, acrylic acid, and 1,2‐propanediol) was dissolved in 0.1 M PB (pH 7.4) to 1000 mM. Hb (15 mg/mL), and each chemical (100 mM) were mixed in PB (pH 7.4) in a total volume of 500 μL and incubated at 37°C for 1–20 days in an orbital shaker‐incubator (ES‐20; Biosan, Riga, Latvia) at 190 rpm (Figure 1a).

### 2.4 Measurement of diHOPrVal production after oral administration of glycidol‐related compounds to mice

Male ICR mice (5 weeks old) were purchased from Japan SLC Corporation (Hamamatsu) and maintained in an animal facility at 23°C ± 1°C with 55% ± 5% humidity under a 12 h light/dark cycle. The mice were fed CE‐2 (Crea Japan). After acclimation for 1 week, glycidol, 3‐MCPD, epichlorohydrin, glyceraldehyde, acrylic acid, and 1,2 propanediol were dissolved in MilliQ water to 0.1 M. Each chemical was administered to the mice orally as a single dose of 1.0 mmol/kg b.w. Dosages in mg/kg units are shown in Table 1. The single dose was administered, and then, the mice were sacrificed by isoflurane inhalation on days 0, 1, 5, 10, and 20 post‐administration of the single dose, and whole blood was collected (Figure 1b). For repeated dose trials, each compound, other than 3‐MCPD and epichlorohydrin, was administered orally daily for 14 days at a dose of 1.0 mmol/kg b.w. 3‐MCPD and epichlorohydrin were given at a concentration of one‐fourth that of the LD_50_. On the 15th day, the animals were sacrificed and whole blood was collected (Figure 1c). Five mice were used for each treatment group and they were provided water ad libitum. Each blood sample (400 μL) was cooled on ice and centrifuged at 1000 × *g* for 5 min. After removing the supernatant plasma, 800 μL of saline was added, the sample was centrifuged at 1000 × g for 5 min, and the supernatant was removed. This procedure was repeated three times to wash the precipitate, which was then stored at −80°C until use. Frozen samples were thawed at room temperature and lysed by adding 150 μL of MilliQ water. The study was approved by the Institutional Animal Care and Use Committees of the university (Permit Number: 215311 and 225,350 date of approval: 30 March 2021 and 22 February 2022).

**TABLE 1:** Dosage and lethal dose 50 (LD_50_) of glycidol‐related compounds.

Chemicals	Single dose (mg/kg b.w.)	Repeat‐dose (mg/kg b.w.)	LD_50_ ^a^ (mg/kg b.w.)
Glycidol	74	74	550^a^
3‐MCPD	110	38	150^a^
Epichlorohydrin	93	49	195^a^
Glyceraldehyde	90	90	3000 (Eng et al., 1972)
Acrylic acid	72	72	1200^a^
1,2‐Propanediol	76	76	20,300^a^

*Note*: Parentheses indicate references cited.

^a^ ChemIDplus [National Institutes of Health, Health & Human Services, https://chem.nlm.nih.gov/chemidplus/rn/. (Accessed on 22 February 2022)].

### 2.5 Dosage information

Our previous study indicated that a single dose of glycidol or glycidol‐related chemicals at 1.0 mmol/kg b.w. in rats produced diHOPrVal (Shimamura et al., 2020). Therefore, for single dosing, each chemical was administered oral gavage at 1.0 mmol/kg b.w. For continuous oral administration over 14 days, only 3‐MCPD and epichlorohydrin, which may cause toxicity at 1.0 mmol/kg b.w, were administered at a concentration of one‐fourth that of the lethal dose 50 (LD_50_). The LD_50_ (Eng et al., 1972), and dose (mg/kg b.w.) for each chemical are listed in Table 1. The human equivalent doses were 6.0 mg/kg b.w. for glycidol, 8.9 mg/kg b.w. for 3‐MCPD (3.0 mg/kg b.w. for repeated dosing), 7.5 mg/kg b.w. for epichlorohydrin (4.0 mg/kg b.w. for repeated dosing), 7.3 mg/kg b.w. for glyceraldehyde, 5.9 mg/kg b.w. for acrylic acid, and 6.2 mg/kg b.w. for 1,2‐propanediol.

### 2.6 FITC derivatization and solid phase extraction

A modified Edman degradation method was used to measure diHOPrVal. For this method, fluorescein‐5‐isothiocyanate (FITC) was used as the Edman reagent to cleave the N‐terminal valine of the chemically modified Hb as a fluorescein thiohydantoin (FTH) derivative and the analyte (glycidol‐Val‐FTH) was measured by LC–MS/MS. The modified Edman degradation method is known as the “Adduct FI*R*E procedure™” because it uses modified Edman degradation to measure the adduct (*R*), which is the product of electrophilic addition reactions using FITC reagents (Rydberg, 2005; Rydberg et al., 2009; Von Stedingk et al., 2010).

The in vitro reaction solutions (500 μL) or blood samples (300 μL) were mixed with 20 μL of 1 M potassium bicarbonate. FITC (5 mg, 13 μmol) dissolved in *N*,*N*‐dimethylformamide (30 μL), internal standard (DHP‐Val‐*d*_7_‐FTH, 100 ppm, 20 μL), and PBS (pH 7.4, 230 μL), followed by incubation at 37°C for 18 h in an orbital shaker‐incubator (ES‐20; Biosan) with shaking (190 rpm). Following FITC derivatization, acetonitrile (1.4 mL) was added to the sample and centrifuged at 14,000 × *g* for 10 min. The supernatant (1.0 mL) was alkalized with 0.5 M ammonium hydroxide (25 μL). Solid phase extraction mixed‐mode anion exchange cartridges (Oasis MAX, 3 cc, 60 mg, Waters Corporation) were preconditioned with 2 mL of acetonitrile. The supernatant was loaded onto the column and washed with 2 mL of acetonitrile, ultrapure water, and 0.5% cyanoacetic acid. The analytes were eluted with 0.25% cyanoacetic acid in acetonitrile (1.4 mL). The solvent was concentrated by nitrogen purging and allowed to dry. The dried sample was dissolved in 10 mL of 0.1% formic acid in acetonitrile: water (1:1, v/v) prior to analysis. The globin concentration in blood was measured using a HemoCue Hb 201^+^ analyzer.

### 2.7 LC–MS/MS measurements

LC–MS/MS was performed using an ACQUITY UPLC system connected to a Devo TQ‐S instrument (Waters Corporation) with an L‐Column2 ODS (2 μm, 2.1 mm × 75 mm; Chemicals Evaluation and Research Institute, Tokyo, Japan) was used. The mobile phase consisted of (A) 0.1% formic acid: acetonitrile (4:1, v/v) and (B) 0.1% formic acid: acetonitrile (1:4, v/v). A gradient for 3 min from 0% B to 50% B, 6 min from 50% B to 100% B, and 4 min of 100% B was used. The injection volume was 2 μL and the flow rate was 0.4 mL/min. For detection, the mass spectrometer conditions were as follows: capillary voltage: 3.00 kV, cone voltage: 30 V, desolvation gas flow: 500 L/h. 500 L/h, cone gas flow: 150 L/h, nebulizer gas flow: 7.0 L/h. Collision energy: 50 eV, and desolvation temperature: 1000°C. After treatment, the samples were analyzed in positive ion mode with multiple reaction monitoring according to the transitions diHOPrVal‐FTH, *m/z* 563 → 390; internal standard DHP‐Val‐*d*_7_‐FTH, *m/z* 570 → 390. The detection limit was set at three times the peak height of the noise. Each target was measured by analyzing a calibration sample of five concentrations containing the internal standard (*r* > .999).

### 2.8 Statistical analysis

The in vivo data were analyzed by a one‐way analysis of variance followed by Dunnett's test using Microsoft Excel 2019 (Microsoft). The significance level was set at *p* < .05 and all experimental results were from five mice per group.

## 3 RESULTS AND DISCUSSION

### 3.1 diHOPrVal production by the reaction of glycidol‐related compounds with human Hb (*in vitro* test system)

**FIGURE 2:**
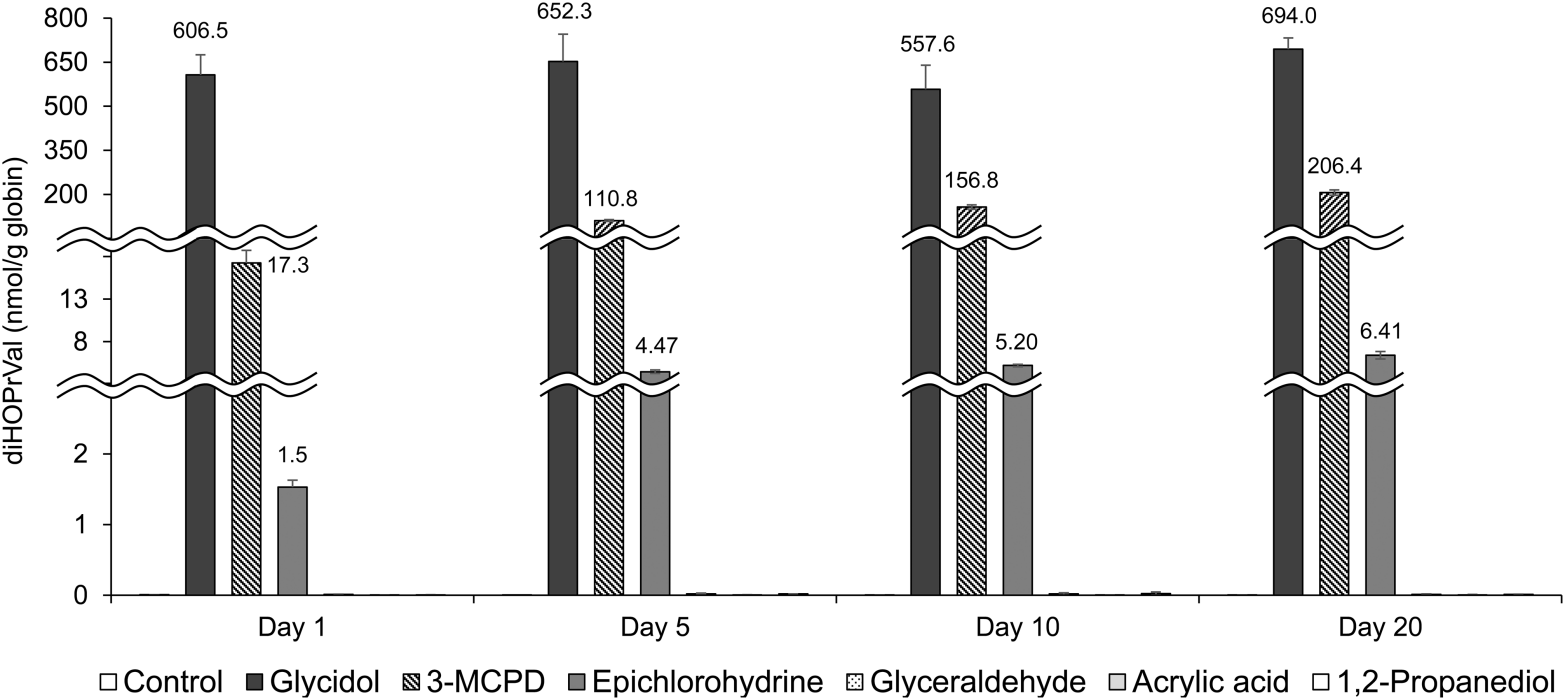
Determination of the amount of glycidol‐hemoglobin (Hb) adducts (diHOPrVal) produced from glycidol‐related chemicals in vitro. Human Hb was incubated with glycidol‐related chemicals at pH 7.4 and 37°C and the amount of diHOPrVal formed was measured after 1, 5, 10, and 20 days. For controls, 0.1 M phosphate buffer (pH 7.4) was used instead of glycidol‐related chemicals.

Human Hb was incubated with glycidol and glycidol‐related compounds for 1, 5, 10, and 20 days at 37°C and the amount of diHOPrVal was measured (Figure 2). On the first day of the reaction, 606.5 ± 68.2 nmol/g globin of diHOPrVal was produced from the reaction with glycidol. The produced amount accounted for approximately 87% of the amount on day 20 of the reaction, which indicates that glycidol produced almost the same level of diHOPrVal over 20 days. This indicates that glycidol binds to the N‐terminal valine of Hb rapidly and binding is maintained.

**FIGURE 3:**
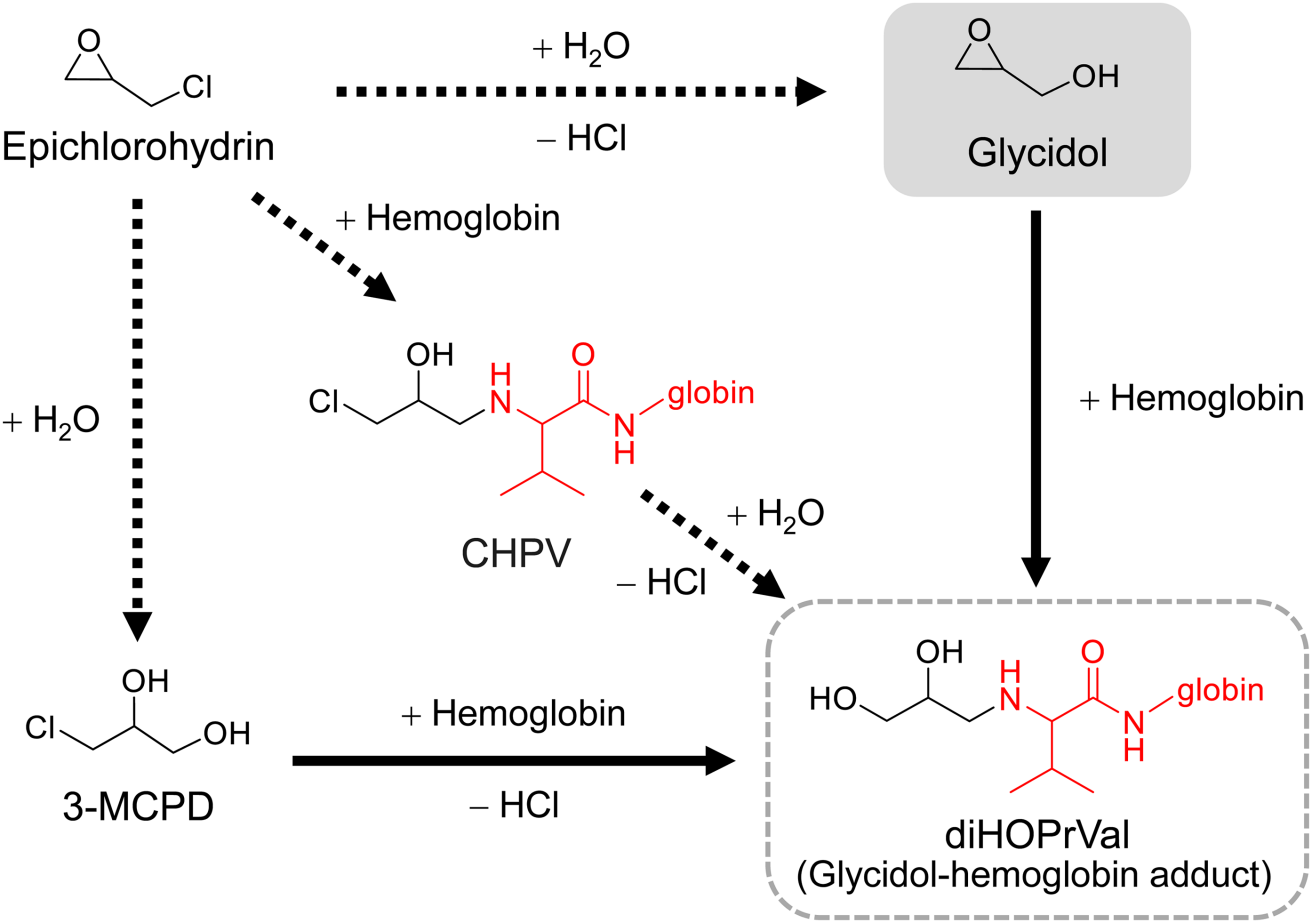
Proposed reaction pathways of epichlorohydrin and 3‐monochloropropane‐1,2‐diol (3‐MCPD) with human hemoglobin (modified according to (Eng et al., 1972)). diHOPrVal; *N*‐2,3‐dihydroxypropyl)valine, CHPV; *N*‐(3‐chloro‐2‐hydroxypropyl)valine.

3‐MCPD and epichlorohydrin, which have been hypothesized to produce diHOPrVal, produced diHOPrVal in smaller amounts compared with an equal molar concentration of glycidol (3‐MCPD: 2.5%–29.7%, epichlorohydrin: 0.2%–0.9%). The amount of diHOPrVal increased in the reaction with 3‐MCPD and epichlorohydrin over time. 3‐MCPD gradually increased the amount of diHOPrVal until day 20 (17.3 ± 1.5 nmol/g globin on day 1, 110.8 ± 3.6 nmol/g globin on day 5, 156.8 ± 6.9 nmol/g globin on day 10, 206.4 ± 9.1 nmol/g globin on day 20). For epichlorohydrin, diHOPrVal levels were unchanged after day 5 (1.53 ± 0.10 nmol/g globin on day 1, 4.27 ± 0.19 nmol/g globin on day 5, 5.20 ± 0.15 nmol/g globin on day 10, 6.41 ± 0.44 nmol/g globin on day 20). *N*‐(3‐chloro‐2‐hydroxypropyl)valine (CHPV) was reported to be an Hb adduct following epichlorohydrin exposure (Bader et al., 2009). A pathway to generate diHOPrVal from CHPV was also proposed (Cocker, 2017). Furthermore, epichlorohydrin may bind to hemoglobin through the conversion of 3‐MCPD to produce diHOPrVal via a proposed pathway (Landin et al., 1996). Epichlorohydrin and 3‐MCPD may take longer to produce diHOPrVal compared with glycidol by the conversion to another compound or via another Hb adduct (such as CHPV) (Figure 3). In addition, for the reaction of epichlorohydrin with water, the half‐life has been reported to be 148 h under neutral, 79 h under acidic, and 62 h under alkaline conditions (Wollin et al., 2014). Therefore, the stability and degradation of chemicals in the reaction solution affected the formation of diHOPrVal. Because the amount of diHOPrVal formed from glyceraldehyde, acrylic acid, and 1,2‐propanediol was similar to that of the control (<0.02 nmol/g globin), it was concluded that diHOPrVal was not formed. Glyceraldehyde is thought to produce diHOPrVal by reduction following the formation of Schiff base adducts (Landin et al., 2000). Acrylic acid also produced acrylic acid‐Hb adducts (*m/z* 561, data not shown), suggesting that diHOPrVal is not generated in vitro. Furthermore, 1,2‐propanediol was not converted to 3‐MCPD in the absence of chloride, suggesting that diHOPrVal is also not formed in vitro.

### 3.2 diHOPrVal production in mice after a single oral administration of glycidol‐related compounds

**FIGURE 4:**
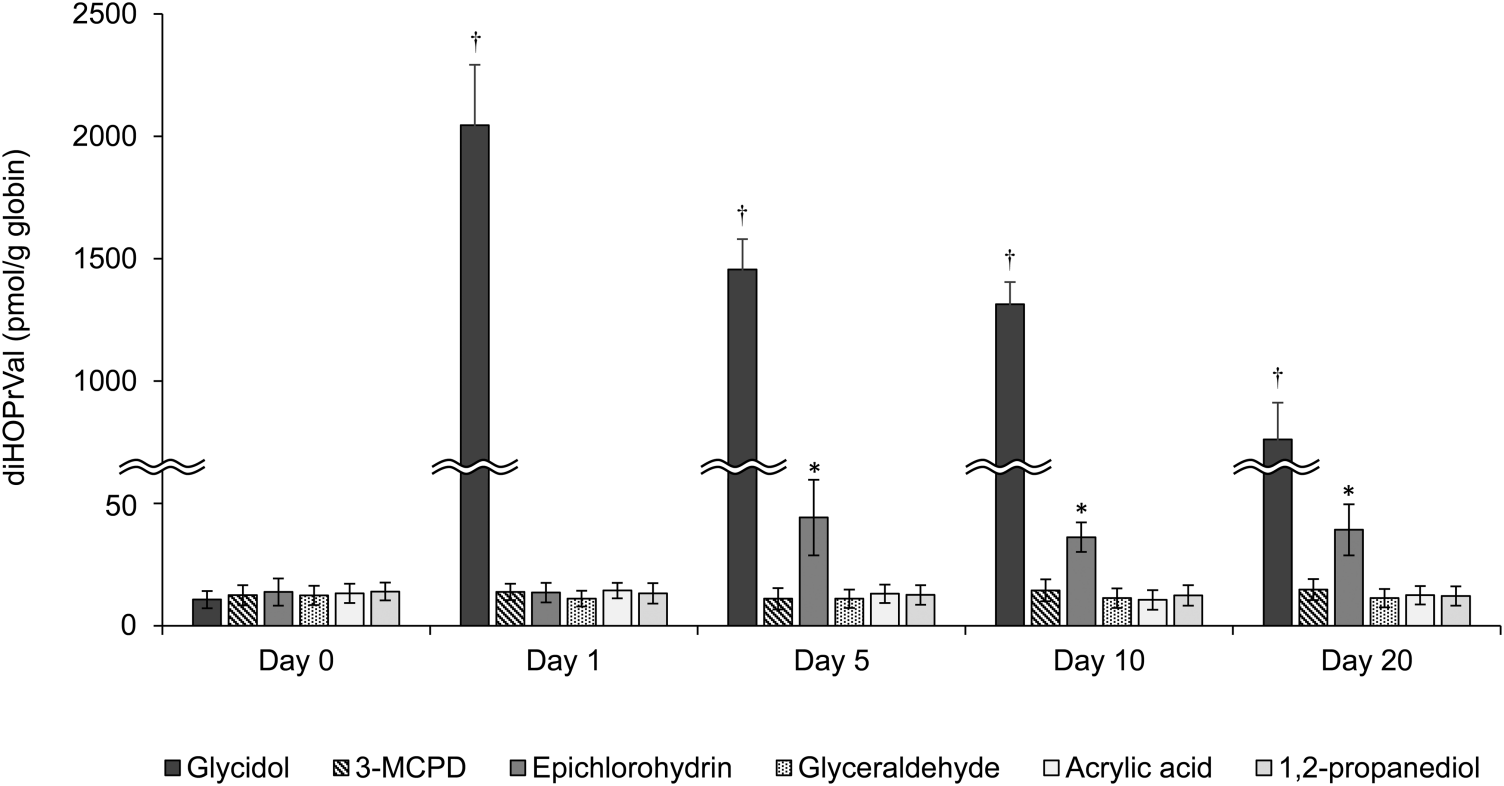
Determination of the amount of glycidol‐hemoglobin (Hb) adduct (diHOPrVal) produced from glycidol‐related chemicals following a single oral administration in vivo. ICR mice were administered a single oral dose of glycidol‐related chemicals and the amount of diHOPrVal formation in blood was measured after 0, 1, 5, 10, and 20 days. †*p* < .05 compared with day 0 of the glycidol‐treated group. *p < .05 compared with the day 0 of the epichlorohydrin‐treated group. The data are presented as the mean ± standard deviation of the results from five mice per group.

**FIGURE 5:**
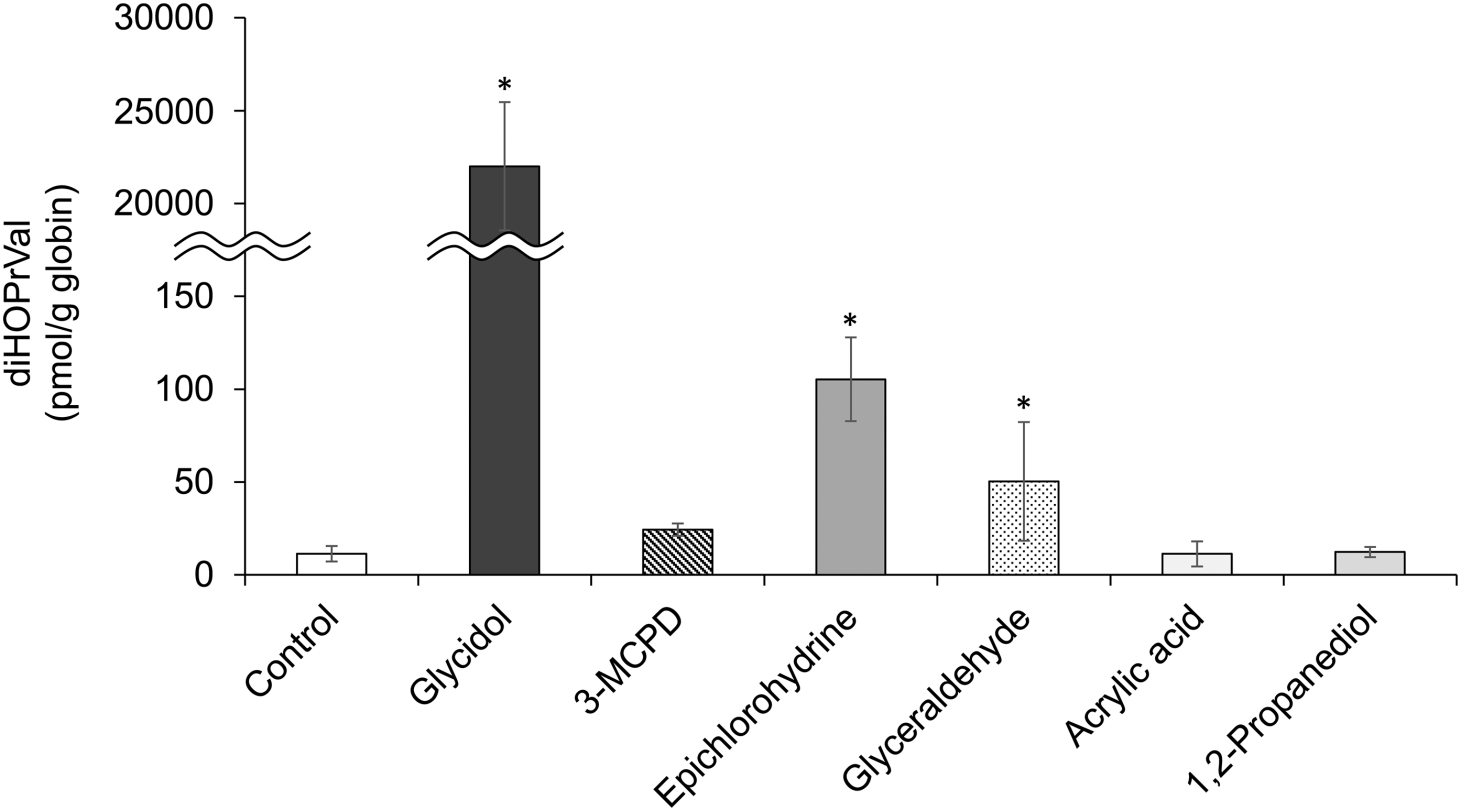
Determination of glycidol‐hemoglobin (Hb) adduct (diHOPrVal) formation by repeated in vivo oral administration of glycidol‐related chemicals. ICR mice were administered repeated oral doses of glycidol‐related chemicals (14 days) and the amount of diHOPrVal produced in the blood was measured 15 days later. *p < .05 compared with the control. The data are presented as the mean ± standard deviation of the results from five mice per group.

The reaction of human Hb and glycidol‐related compounds was examined in vitro to determine whether the Hb adduct diHOPrVal is formed. However, to determine the risk of these chemicals to humans and the role of Hb adducts as exposure indicators, it is necessary to verify the results using experimental animals. Therefore, next, an in vivo test system was utilized to confirm the formation of Hb adducts from glycidol‐related compounds observed in vitro. ICR mice were administered a single oral dose of a glycidol‐related compound and the amount of Hb adducts in mouse blood was measured 1–20 days after administration. The results showed that diHOPrVal was detected only in the glycidol‐ and epichlorohydrin‐treated groups at a single dose (Figure 4). In the glycidol administration group, diHOPrVal production was the highest on day 1 following administration (2046 ± 247 pmol/g globin) and it gradually decreased over time (Figure 5). Unstable Hb adducts are rapidly eliminated because of simultaneous metabolism and first‐order chemical instability. In contrast, stable Hb adducts disappear by zero‐order kinetics that are defined by erythrocyte lifetime (Fennell et al., 1992; Troester et al., 2001). In the glycidol‐treated group, in which there were no hematologic or other toxicities after a single exposure, the amount of diHOPrVal decreased linearly with erythrocyte lifespan.

In the epichlorohydrin‐treated group, diHOPrVal was not detected on day 1 following administration; however, 36–44 pmol/g globin was produced 5–20 days later (Figure 4). In vitro, diHOPrVal production from 3‐MCPD was greater than 10‐fold higher compared with that of epichlorohydrin at equimolar amounts (Figure 2). In contrast, after a single dose in vivo, 3‐MCPD did not produce any diHOPrVal, whereas diHOPrVal production from epichlorohydrin was approximately 1/50th that of glycidol. 3‐MCPD is rapidly and completely absorbed from the gastrointestinal tract, widely distributed in the body, and excreted in a metabolized form through the urine and by respiration (Abraham et al., 2021; Bergau et al., 2021). This suggests that 3‐MCPD may be more readily metabolized and excreted in vivo as diHOPrVal was not produced. The chemical stability of diHOPrVal produced from epichlorohydrin has been previously reported in vivo (Landin et al., 1999), which is consistent with the results of the present study. DiHOPrVal was not produced from glyceraldehyde, acrylic acid, or 1,2‐propanediol as well as from 3‐MCPD, in which diHOPrVal was detected in vitro.

### 3.3 diHOPrVal production in mice after repeated oral administration of glycidol‐related compounds

**FIGURE 6:**
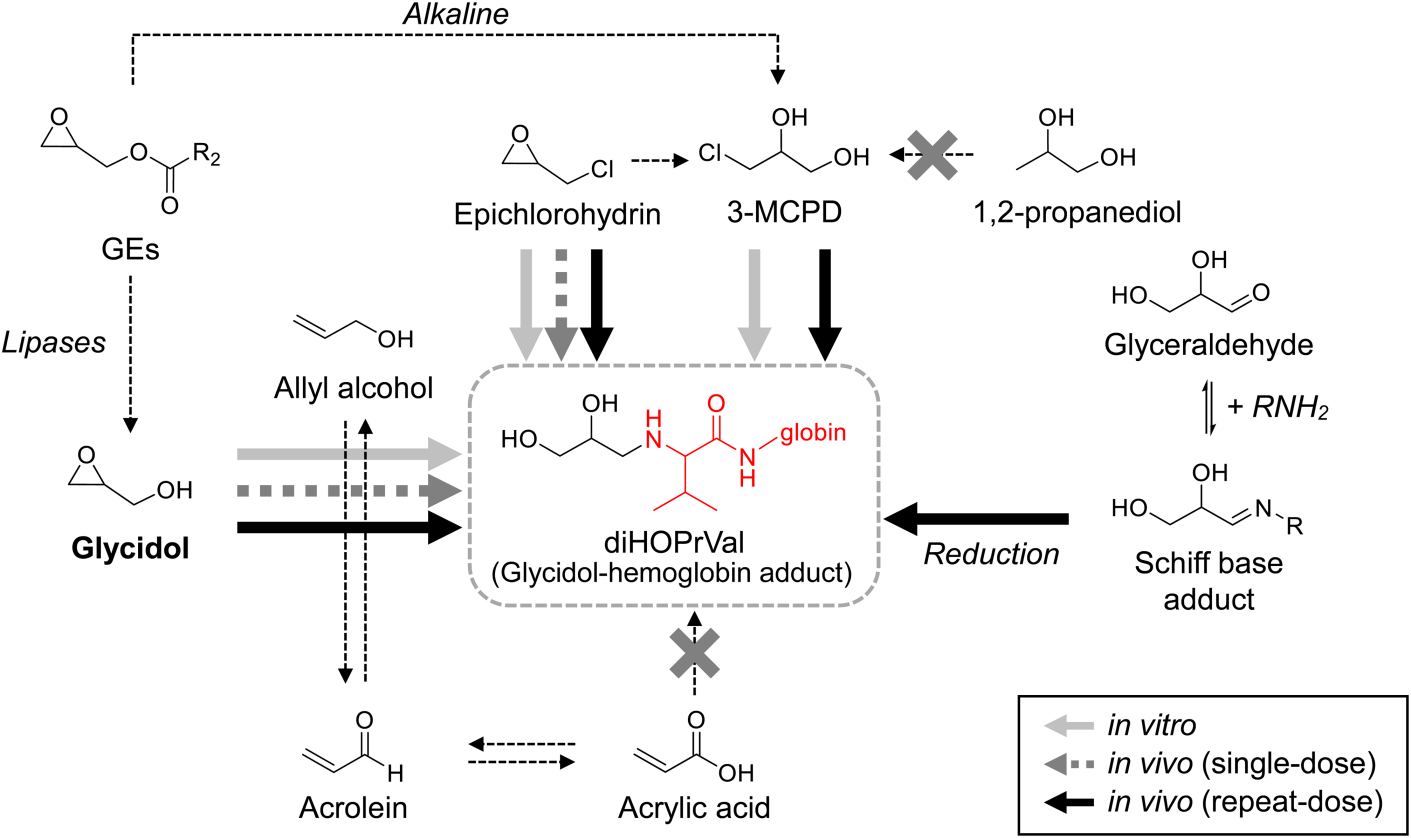
Glycidol‐related chemicals that produce a glycidol‐hemoglobin adduct (diHOPrVal).

After the oral administration of glycidol‐related compounds to ICR mice for 14 consecutive days, the amount of diHOPrVal in mouse blood collected on day 15 was measured (Figure 5). The results indicated that 22,001 ± 3458 pmol/g globin of diHOPrVal was present in the glycidol‐treated group, an increase of more than 10‐fold compared with the single dose group. In the present study, diHOPrVal, the Hb adduct of glycidol, was generated from various glycidol‐related chemicals (3‐MCPD, epichlorohydrin, and glyceraldehyde) following repeated oral administration in vivo (Figure 6). In the epichlorohydrin‐treated group, 105 ± 23 pmol/g globin of diHOPrVal was detected, which was approximately 4‐fold higher compared with the single dose. 3‐MCPD, in which diHOPrVal was not detected in the single dose, 24 ± 3.4 pmol/g globin was produced following repeated dosing (Figure 4). The amounts of diHOPrVal produced by epichlorohydrin and 3‐MCPD in vivo were <1% of diHOPrVal produced by an equal molar concentration of glycidol (epichlorohydrin: 0.17%–0.31%, 3‐MCPD: 0.017%–0.031%). In the present study, a single in vivo dose did not result in the formation of diHOPrVal from 3‐MCPD (Figure 4). In addition to 3‐MCPD being readily metabolized in vivo (Abraham et al., 2021; Bergau et al., 2021). Aasa et al. found that 3‐MCPD is approximately 1000‐fold less reactive with the N‐terminal valine of Hb compared with glycidol (Cheng et al., 2017). Even with repeated dosing, the amount of diHOPrVal produced from 3‐MCPD was negligible, which was consistent with our results. Glyceraldehyde, which did not produce diHOPrVal in vitro or following a single dose in vivo, produced 50 ± 32 pmol/g globin of diHOPrVal (0.10%–0.34% of an equal molar concentration of glycidol) after repeated doses (Figure 5). The production of diHOPrVal from glyceraldehyde requires the formation of a Schiff base at the N‐terminal valine of Hb followed by reduction to produce diHOPrVal (Landin et al., 1999). Hb adducts from Schiff bases may be reduced in vivo, because the reduced Schiff base adduct [N‐(4‐hydroxybenzyl)valine Hb adduct] was detected when 4‐hydroxybenzaldehyde was incubated overnight with fresh whole blood (Degner et al., 2018). In our previous study, there was no correlation between the amount of diHOPrVal and exposure to GEs, the precursor of glycidol, which suggests that we are routinely exposed to chemicals other than GEs. However, the amount of diHOPrVal formed from chemicals other than glycidol was less than 1/100 of that from glycidol and GEs. These results suggest that various glycidol‐related chemicals (e.g., 3‐MCPD, epichlorohydrin, and glyceraldehyde) are not likely to have a significant effect on diHOPrVal formation.

In this study, mice were exposed to single chemicals; however, a variety of chemicals coexist in the food and the environment. Thus, various Hb adducts derived from many chemicals have been found in human blood (Carlsson et al., 2014; Degner et al., 2018; Goel et al., 2013). Other Hb adducts have been identified from substances other than glycidol, including ethylene oxide, glyoxal, methyl vinyl ketone, acrylamide, acrylic acid, methyl glyoxal, ethyl vinyl ketone, glycidamide (Carlsson et al., 2019), and furfuryl alcohol (Monien et al., 2021). Glycidol administration to mice or rats resulted in the formation of a hydroxypropanoic acid valine adduct (*m/z* 577) from glycidic acid, a minor metabolite of glycidol (Vryonidis et al., 2022). Furthermore, our previous study showed that acrylamide or glucose can affect the formation of diHOPrVal (Shimamura et al., 2022). Thus, the effect of combined exposure to various chemicals in food and the environment may also affect the formation of diHOPrVal, a marker of glycidol.

Although this study confirmed once again the usefulness of diHOPrVal as a marker for glycidol, it is important to consider the contribution of other related chemicals with different biological behaviors to diHOPrVal formation for validating the results and interpreting unexpected results, particularly, the in vivo metabolism of each chemical. In the present study, 3‐MCPD produced large amounts of diHOPrVal in vitro, but not in vivo. Therefore, it is expected that even chemicals that produce diHOPrVal because of their original chemical structure may be less likely to produce diHOPrVal in vivo due to metabolism or excretion. Further studies are needed to determine the effects of various chemicals in vivo and on the formation of diHOPrVal.

## AUTHOR CONTRIBUTIONS

**Yuko Shimamura:** Data curation (lead); formal analysis (equal); writing – original draft (equal). **Yuri Wada:** Formal analysis (equal). **Moeka Tashiro:** Formal analysis (equal). **Hiroshi Honda:** Data curation (equal); methodology (equal); writing – review and editing (equal). **Shuichi Masuda:** Data curation (equal); methodology (equal); project administration (lead); writing – review and editing (equal).

## CONFLICT OF INTEREST STATEMENT

Although H.H. is an employee of Kao Corporation, which provided funding for this work, this study was conducted for non‐profit purposes. No inappropriate data have been created based on the existence of a financial relationship with a company.

## Data Availability

The data that support the findings of this study are available from the corresponding author upon reasonable request.
